# Chain formation can enhance the vertical migration of phytoplankton through turbulence

**DOI:** 10.1126/sciadv.aaw7879

**Published:** 2019-10-16

**Authors:** Salvatore Lovecchio, Eric Climent, Roman Stocker, William M. Durham

**Affiliations:** 1 Institut de Mécanique des Fluides (IMFT), Université de Toulouse, CNRS, Allée du Professeur Camille Soula, 31400 Toulouse, France.; 2 Institute for Environmental Engineering, Department of Civil, Environmental and Geomatic Engineering, Eidgenössische Technische Hochschule (ETH) Zürich, 8093 Zurich, Switzerland.; 3 Department of Zoology, South Parks Road, University of Oxford, Oxford OX1 3PS, UK.; 4 Department of Physics and Astronomy, University of Sheffield, Hounsfield Road, Sheffield S3 7RH, UK.

## Abstract

Many species of motile phytoplankton can actively form long multicellular chains by remaining attached to one another after cell division. While chains swim more rapidly than single cells of the same species, chain formation also markedly reduces phytoplankton’s ability to maintain their bearing. This suggests that turbulence, which acts to randomize swimming direction, could sharply attenuate a chain’s ability to migrate between well-lit surface waters during the day and deeper nutrient-rich waters at night. Here, we use numerical models to investigate how chain formation affects the migration of phytoplankton through a turbulent water column. Unexpectedly, we find that the elongated shape of chains helps them travel through weak to moderate turbulence much more effectively than single cells, and isolate the physical processes that confer chains this ability. Our findings provide a new mechanistic understanding of how turbulence can select for phytoplankton with elongated morphologies and may help explain why turbulence triggers chain formation.

## Teaser

The elongated shape of multicellular phytoplankton chains helps them migrate through turbulence.

## INTRODUCTION

The remarkable diversity of phytoplankton morphologies found in a single drop of water has been a source of fascination since the advent of the modern microscope (*1*). The evolutionary pressures that underlie phytoplankton shape are likewise diverse, affecting a wide range of important processes, including nutrient acquisition, light capture, sedimentation rate, predation, and motility (*2*–*9*). While most phytoplankton species maintain a relatively uniform shape over time, others actively alter their size and shape by growing spines (*10*), aggregating into clumps (*11*), or forming chains. Chain formation is widely studied because it occurs in a diverse range of phytoplankton species, including cyanobacteria (*12*), diatoms (*7*, *9*), and dinoflagellates (*2*, *3*, *8*, *13*–*17*). Chains form when daughter cells remain attached to one another after cell division, creating highly elongated structures. Larger predators like copepods preferentially graze on chains, and consequently, the chemical secretions from copepods induce chains to break apart, demonstrating that phytoplankton can actively regulate chain length (*3*, *7*, *9*).

Despite their relatively small size (∼1 to 1000 μm), motile phytoplankton can traverse tens of meters of the water column per day, moving from the well-lit surface waters during the day to depth at night, where nutrients are more abundant and predation risk is reduced (*18*). Chain formation in motile phytoplankton has long been associated with increased swimming speed. Swimming speed increases with chain length because chain formation increases propulsive thrust more than it does hydrodynamic drag (see Materials and Methods), and this allows chains to swim 50 to 200% faster than single cells (*3*, *6*, *8*, *19*). However, these measurements are from quiescent laboratory conditions, which do not provide a complete picture of the effect of chain formation on migration. In nature, turbulence exerts hydrodynamic torques on phytoplankton and can thereby randomly reorient their swimming direction, inhibiting their ability to vertically migrate (*20*). In turbulence, single spherical cells and elongated chains experience different viscous torques: While vorticity generates a torque on both types of cells that tends to rotate them end over end, elongated cells also experience a further torque that tends to align them in the direction of principal strain. It is not known, however, how this difference in torques affects vertical migration. Here, we embed an individual-based model of phytoplankton chain motility into a direct numerical simulation (DNS) of turbulence to understand how chain formation affects vertical migration through a turbulent seascape. We find that chain formation enhances vertical migration across a wide range of ecologically relevant conditions and resolve the physical mechanisms that are responsible.

## RESULTS

### Quantifying vertical migration in turbulent flows

Turbulence exerts viscous torques on phytoplankton cells, and these torques act to reorient cell motility in random directions. To maintain their orientation in the face of these viscous torques, many motile species benefit from a stabilizing torque that acts to keep their motility oriented in the vertical direction. When a cell’s swimming direction is under the control of both viscous and stabilizing torques, it is said to be gyrotactic (*21*). Stabilizing torques can arise from a number of different mechanisms, including inhomogeneity in cell density and cell morphology, and, potentially, flagellar movement (*21*–*23*). Regardless of the specific mechanism, a cell’s overall stability can be quantified in terms of a gyrotactic reorientation time scale, *B*, which measures how long it takes for a cell perturbed from its equilibrium orientation to return to it in the absence of flow (*24*).

The swimming direction of a spherical cell is only affected by the ambient fluid flow through the fluid’s vorticity, **ω**, whereas elongated cells experience an additional torque that arises from the fluid’s rate of strain, **E**. More formally, changes in the swimming direction **p** of a cell that swims along its long axis are predicted by

$$\frac{dp}{\mathrm{dt}}=\frac{1}{2\Psi }[k-(k\cdot p)p]+\frac{1}{2}\omega \times p+\alpha [I-pp]\cdot E\cdot p$$ (1)

where Ψ = *B*ω_K_ is a nondimensional stability number, **k** is the vertical direction in which a cell would swim in the absence of flow, α = (*r*^2^ − 1)/(*r*^2^ + 1) is the cell eccentricity, *r* is the cell aspect ratio, ω_K_ = (ε/ν)^1/2^ is the magnitude of the vorticity at the Kolmogorov scale, ε is the rate of turbulent energy dissipation, and ν is the fluid’s kinematic viscosity (*20*, *24*–*26*). This parameterization assumes that cells can be approximated as prolate spheroids whose aspect ratio *r* is given by their length divided by their width. The first term on the right-hand side of this equation models the stabilizing torque, the second term models the tendency for vorticity to overturn the cell, while the last term models the tendency for elongated cells to align with the direction of principal strain. For spherical cells (*r* = 1), the last term vanishes, but not for elongated chains, for which we can assume that *r* is proportional to the number of cells in the chain, *n*. Cell trajectories, **x**(*t*), are determined by integrating the total cell velocity, i.e., the sum of the swimming velocity, *V*_C_**p**, and the flow velocity at the cell location, **u**, such that

dx/dt=Φp+u (2)

where time is nondimensionalized by the Kolmogorov time scale 1/ω_K_ and length by the Kolmogorov length scale η_K_ = ν^3/4^ε^−1/4^. Here, **u** is the nondimensional flow velocity, which is rescaled by the Kolmogorov velocity scale *V*_K_ = ω_K_η_K_ = (νε)^1/4^. Thus, for spherical cells, two dimensionless parameters—the stability number Ψ and the swimming number Φ = *V*_C_/*V*_K_—fully parameterize the process of swimming in turbulence, whereas for chains, a third dimensionless parameter arises, the eccentricity α, which quantifies chain morphology (*20*, *25*, *26*).

**Fig. 1:**
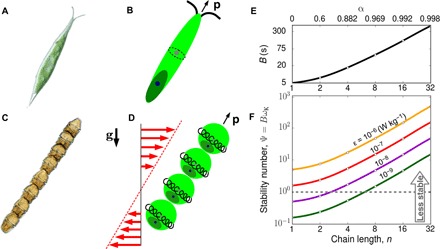
Both single phytoplankton cells and multicellular phytoplankton chains have elongated shapes, which can markedly affect their ability to migrate through turbulence. (**A**) The unicellular *Chlorogonium elongatum* displays a highly elongated morphology, a characteristic of many species of motile phytoplankton (*2*). (**B**) The center of mass of unicellular phytoplankton (blue circle) can be separated from their center of buoyancy (gray circle), for example, because dense organelles are asymmetrically distributed within their bodies (*21*). This gives rise to a stabilizing torque that acts to keep the direction of motility **p** pointed in the vertical direction and facilitates migration over the depth of the water column. (**C**) Chain-forming species, such as the eight-cell chain of the dinoflagellate *Gymnodinium catenatum* shown here, also exhibit a highly elongated morphology. (**D**) By analyzing the balance of torques on the idealized chain shown here, we predict that when longer chains are perturbed away from their equilibrium orientations, they require a much larger characteristic time scale, *B*, to return than that of a single cell (Materials and Methods). (**E**) The gyrotactic reorientation time scale, *B*, increases with chain length. While single cells have reorientation time scales of order *B* = 5 s, a hydrodynamic model predicts that the *B* of a chain eight cells long is an order of magnitude greater. (**F**) The nondimensional parameter Ψ = *B*ω_K_ measures the stability of a cell relative to the Kolmogorov scale shear, ω_K_, that they experience in turbulent environments. When Ψ ≳ 1, vertical motility is expected to be suppressed (*20*). Thus, based purely on stability considerations, chains larger than eight cells are expected to lack the ability to migrate through even relatively weak turbulence (i.e., with a dissipation rate of ε = 10^−9^ W/kg). The cell shown in (A) is approximately 100 μm long and is reproduced with permission of Y. Tsukii (Hosei University, Japan). The chain shown in (C) is approximately 300 μm long and is reproduced with permission of H. Chang (National Institute of Water and Atmospheric Research, New Zealand).

To understand how chain formation affects phytoplankton stability and therefore overall migration rate, we first consider how chain formation affects cell orientation in the absence of flow. While a number of different factors can affect cell stability (*21*–*23*), the reorientation time scale is often computed as an effective bottom heaviness (*24*), *B* = μα_⊥_/(2*h*ρ*g*), where μ is the dynamic viscosity of the fluid, *h* is the distance between the cell’s center of mass and its center of buoyancy, ρ is the cell density, *g* is gravity, and α_⊥_ is a dimensionless coefficient that measures the viscous resistance of a prolate spheroid to rotation in a direction orthogonal to **p** (Supplementary Text). As a chain increases in length, the distance from its center of mass to its center of buoyancy remains the same as that of one of its constituent cells, i.e., *h* is independent of chain length (Materials and Methods). In contrast, α_⊥_, and thus the viscous torque that resists stabilization, increases with chain length. Consequently, in quiescent conditions, longer chains will require more time (larger *B*) to reach their equilibrium orientation if perturbed away from it. For example, an eight-cell chain will reorient approximately 10 times more slowly than one of its constituent cells swimming on its own (Fig. 1E).

Previous studies found that the stability number, Ψ, is a good predictor of a spherical cell’s capacity to vertically migrate through both laminar and turbulent flows (*20*, *27*, *28*). In steady laminar flows, spherical cells are overturned when *B*ω > 1 and vertical migration is altogether arrested (*28*). In turbulent flow, where the relevant velocity gradients occur at the Kolmogorov scale, the vertical migration rate of spherical cells is unaffected when Ψ = *B*ω_K_ ≲ 1 but sharply decreases (because turbulence randomly reorients cells) as Ψ increases. For example, for Ψ = 10, the rate of vertical migration is approximately 20% of the cell swimming speed (*20*). If the same paradigm holds for chains, we would expect that even relatively weak turbulence would markedly impede their vertical migration, in light of their much-reduced intrinsic stability (i.e., larger *B*; Fig. 1F). However, this prediction stands in contrast with field observations that show that chains routinely traverse turbulent water columns (*2*, *15*), suggesting that the interaction of chains with small-scale turbulence has unexpected dynamics.

Here, we use a numerical model to demonstrate that the elongated shape of phytoplankton chains has a profound impact on their ability to migrate through turbulence. We use an individual-based model to simulate the movement of a large number of gyrotactic swimmers as they negotiate a realistic turbulent flow. Isotropic turbulence is obtained via DNS by integrating the full Navier-Stokes equations within a triply periodic domain. This technique injects energy into the system at large spatial scales, and this energy then cascades down to the Kolmogorov scale where it is dissipated by viscosity (Materials and Methods). Once the flow reaches statistical steady state, it is seeded with 100,000 swimmers having random initial position and orientation. Organism orientations and trajectories are computed using Eqs. 1 and 2, and the resulting data are analyzed after the distribution of swimmers has reached a statistical steady state (Materials and Methods).

### Elongation helps chains keep their motility directed along the vertical

Chain formation decreases orientational stability (Fig. 1E), increases swimming speed (*3*, *6*, *8*, *19*), and causes swimmers to be reoriented not only by vorticity but also by hydrodynamic strain (Eq. 1). Each of these three processes can affect rates of vertical migration. To understand the effect of cell shape on migration, we first consider swimmers with a fixed stability number, Ψ, and swimming number, Φ, and vary only their elongation α (α = 0 for spheres, α ≈ 1 for needles). Chain formation occurs via cell division, which is often synchronized so that chains of 2, 4, 8, 16, and 32 cells are most frequently observed (*3*, *15*, *17*, *19*). The contribution of the rate of strain (proportional to α; Eq. 1) on swimmer orientation rapidly saturates with chain length *n*, increasing from α = 0.600 to 0.882 to 0.969 when chains increase in length from *n* = 2 to 4 to 8 cells, respectively (Fig. 1E). Therefore, the limit α = 1 provides a good approximation for longer chains.

**Fig. 2:**
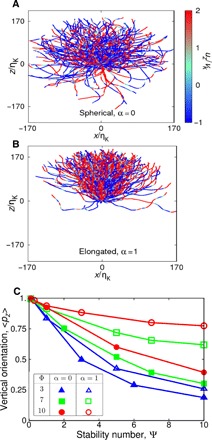
Elongation stabilizes the orientation of cells in turbulent flow, allowing them to keep a larger fraction of their motility oriented in the vertical direction. (**A** and **B**) Here, we plot the trajectories of 200 single spherical cells [α = 0 (A)] and highly elongated chains [α = 1 (B)] that are swimming in a DNS of turbulence. While chain formation can affect both the intrinsic stability (Fig. 1) and the swimming speed (Fig. 4A), here, we use [Ψ = 10, Φ = 10] in both simulations to isolate the effect of swimmer shape. As advection by flow adds considerable noise, the trajectories shown here have been postprocessed to show movement only due to motility (*d***x**/*dt* = Φ**p**). Trajectories show movement over 20 Kolmogorov time scales ($20\omega_{K}^{-1}$) and have been color-coded to reflect the instantaneous vertical fluid velocity, *u_z_*/*V*_K_, at the cell location. While these trajectories occur at different positions within our three-dimensional computational domain, for the purposes of presentation, we have taken the [*x*, *z*] projection of each trajectory and moved its initial position to the origin. (**C**) To more directly quantify how elongation affects swimming direction in turbulence, we measured the mean vertical projection of the swimming direction, 〈*p_z_*〉, for 100,000 swimmers with either spherical (filled symbols) or highly elongated (open symbols) morphologies. A population of cells that swims strictly upward against gravity yields 〈*p_z_*〉 = 1, while 〈*p_z_*〉 = 0 for a population of cells that move in random directions. This analysis shows that elongation can substantially enhance the stability of cells, and this effect becomes more pronounced as cell speed, Φ, increases.

First, to obtain a qualitative picture of the effect of elongation, we computed trajectories of both single spherical cells (α = 0) and chains (α = 1), removing the contribution of fluid advection in postprocessing to isolate the cell’s movement relative to the flow (Fig. 2, A and B). Chains spend a greater proportion of time moving in the vertical direction compared to single cells, suggesting that, all other factors being equal, elongation enhances vertical migration through turbulence. This conclusion is also borne out in quantitative measurements of the average vertical projection of a population’s swimming direction, 〈*p_z_*〉 (Fig. 2C). While elongation has a negligible effect on chains with a strong stabilizing torque (Ψ < 0.1), it can profoundly enhance the ability of weakly stable chains to keep their motility directed along the vertical direction. This acts to compensate for the loss of intrinsic stability caused by chain formation (Fig. 1, E and F). Furthermore, elongation is more effective at stabilizing the vertical orientation the faster a chain swims: for weakly stable chains (Ψ = 10), elongation increases 〈*p_z_*〉 by 38% for a chain swimming at Φ = 3. However, if the same chain swims approximately three times faster (Φ = 10), elongation increases 〈*p_z_*〉 by 96%. Our findings thus suggest that the increased swimming speed of chains pushes them into a regime where their elongated shape is more effective in keeping their motility oriented in the vertical direction.

### Chains can preferentially accumulate in regions of flow that propel them faster along their migration direction

**Fig. 3:**
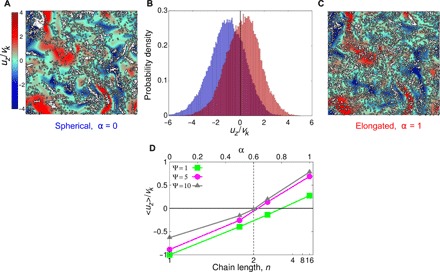
Single spherical cells accumulate where they have to swim against the flow, while elongated chains accumulate where flow propels them in the same direction as their motility. (**A** to **C**) Two simulations were performed with identical motility parameters [Ψ = 1, Φ = 10], except that in one simulation, cells were spherical α = 0, whereas in the other, they were highly elongated, α = 1. (A) and (C) show a horizontal slice through the three-dimensional simulations, in which the white circles show cell positions and the color map shows the vertical fluid velocity, *u_z_*, normalized by the Kolmogorov velocity, *V*_K_. (B) We measured *u_z_* at the location of 100,000 cells to quantify how shape affects the vertical transport of cells by flow. Upward-migrating spherical cells (blue) tend to accumulate in regions of downwelling (*u_z_* < 0), whereas upward-migrating elongated cells (red) tend to accumulate in regions of upwelling (*u_z_* > 0). (**D**) To understand how these dynamics vary with chain length, we calculated the mean vertical fluid velocity at the location of swimmers with different chain lengths. We find that as chains get longer, they progressively move from downwelling to upwelling regions of the flow, suggesting that longer chains benefit from this mechanism more than shorter ones. Similar trends were observed for different values of the nondimensional stability number, Ψ, although the transition to upwelling occurs at larger chain lengths for more stable cells (i.e., for smaller Ψ). All simulations used a nondimensional swimming speed Φ = 10.

**Fig. 4:**
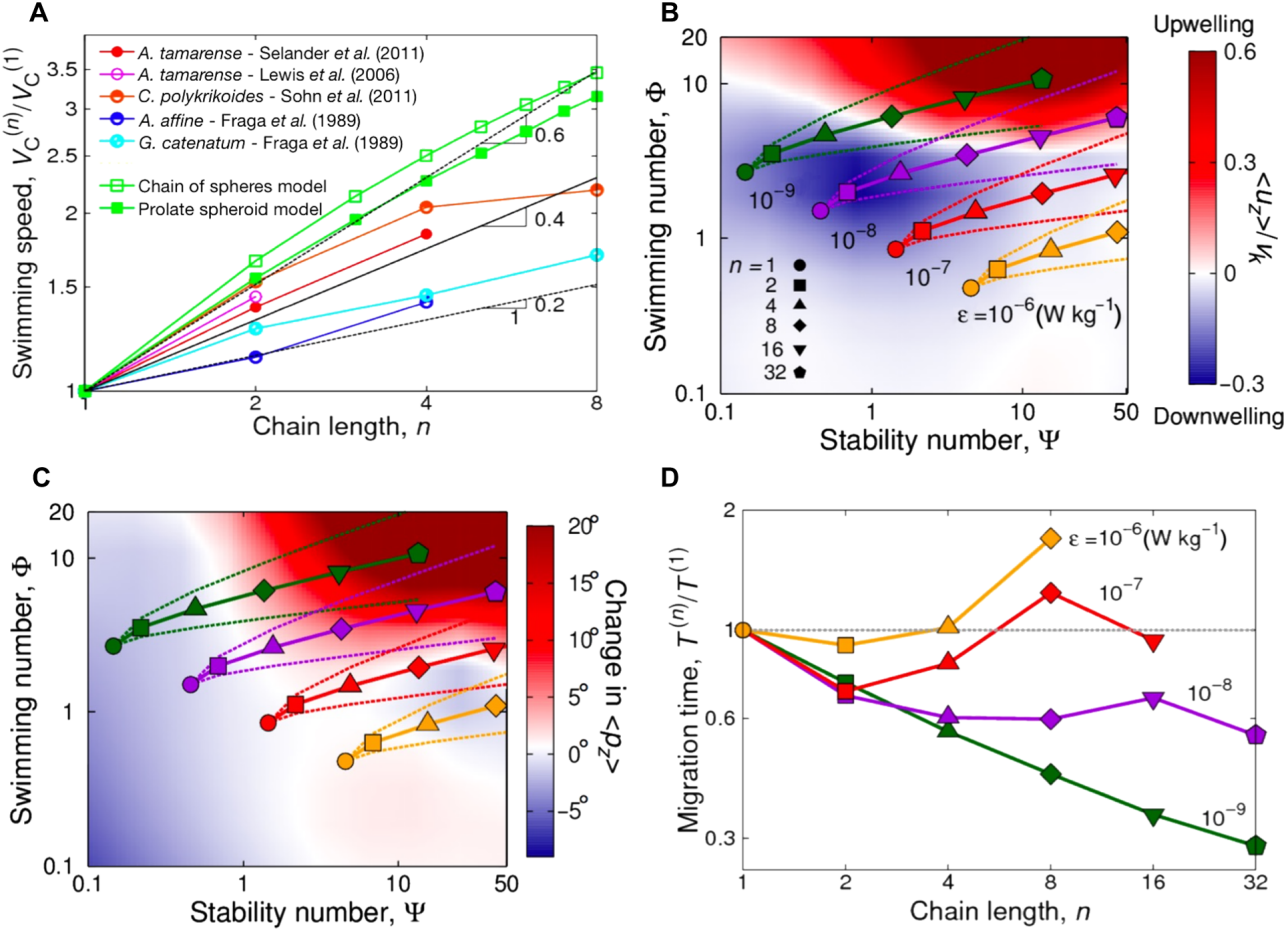
Chain formation allows motile phytoplankton to migrate faster through relatively weak turbulence. (**A**) Empirical measurements show that swimming speed increases with chain length, a finding that is consistent with models that assume that thrust increases at a faster rate than hydrodynamic drag as a chain gets longer (green symbols; Materials and Methods). While there is variability across different species, these results suggest that the swimming speed of a chain of length *n* can be approximated using $V_{C}^{\left(n\right)}/V_{C}^{\left(1\right)}∼n^{\beta }$, where $V_{C}^{\left(1\right)}$ is the swimming speed of a single cell and β is an exponent with a value on the interval [0.2 0.6]. We note that we omitted (*16*) from this analysis because they only reported swimming speeds projected along a single axis. (**B** and **C**) We combine a model that estimates how the gyrotactic orientation time scale *B*^(*n*)^ changes with chain length (Fig. 1E) with our expression for $V_{C}^{\left(n\right)}$ to understand the [Ψ, Φ] parameter space sampled by a chain as it increases in length and experiences different turbulent dissipation rates, ε. Here, we assume that single cells have *B*^(1)^ = 5 s and $V_{C}^{\left(1\right)}=500$ μm/s, which are representative values from the literature (*2*, *21*–*23*, *28*). Solid lines show results for β = 0.4, while dashed lines show β = 0.2 and 0.6 to illustrate potential variability in Φ (lower and upper dashed lines, respectively). The color map in (B) shows 〈*u_z_*〉/*v*_K_, the mean vertical fluid velocity at the centroid of swimmers (α = 1), which indicates that chain formation in relatively weak turbulence (ε = 10^−9^ to 10^−8^ W/kg) induces a shift from accumulating in downwelling parts of the flow 〈*u_z_*〉 < 0 to accumulating in upwelling parts of the flow 〈*u_z_*〉 > 0. However, in stronger turbulence (ε > 10^−7^ W/kg), both single cells and chains accumulate in downwelling regions. The color map in (C) shows the change in the average swimming direction 〈*p_z_*〉 caused by elongation, calculated here as the difference in 〈*p_z_*〉 between highly elongated chains (α = 1) and spherical single cells (α = 0). This indicates that chain formation enhances the ability of cells to keep their motility pointed in the vertical direction in all but the most intense turbulence (i.e., as long as ε < 10^−6^ W/kg). (**D**) Using both our model for Ψ (Fig. 1F) and our fit of empirical data for Φ [(**A**), β = 0.4], we estimate how chain formation affects the overall migration rate of cells at different turbulent dissipation rates. We normalized *T*^(*n*)^, the time required for a chain to traverse a fixed distance through a turbulent water column, by *T*^(1)^, the time needed by a single cell to swim the same distance. Thus, if *T*^(*n*)^/*T*^(1)^ is smaller than unity, chains can migrate through turbulence more quickly than single cells. This analysis indicates that chain formation markedly enhances vertical migration in relatively weak turbulence. However, in stronger turbulence, chain formation can either increase or decrease migration time, depending on the length of the chain.

While many phytoplankton swim multiple body lengths per second (*2*), their transport is also affected by ambient flows in their environment. Motile phytoplankton do not sample a flowing environment uniformly, but rather, the interaction between stabilizing and hydrodynamic torques causes them to swim toward specific regions of the flow, and this, in turn, has a major effect on their net transport. For example, the torques arising from vorticity have been shown to reorient upward-swimming unicellular phytoplankton toward downwelling regions in both simple flows, like Poiseuille flow through a tube, and more complex flows, like turbulence (*20*, *21*, *24*, *26*, *29*). In contrast, hydrodynamic strain can cause elongated swimmers in turbulence to move toward regions of the flow moving in the same direction as their vertical migration, provided that they swim sufficiently quickly (Figs. 3 and 4B) (*26*, *29*), propelling them faster in the direction of their intended migration. Similar to previous works that study elongated motile cells (*25*, *26*, *29*–*31*), we find that elongation can profoundly affect how swimmers cluster in turbulence (figs. S1 and S2). Relatively little attention, however, has been given to understanding how elongation affects phytoplankton’s ability to vertically migrate through a turbulent water column.

To illustrate how chain formation can alter flow-mediated vertical transport, we exposed both spherical (α = 0) and elongated swimmers (α = 1) with identical Ψ and Φ values to the same turbulent flow (Fig. 3, A and C). While gyrotaxis acts to reorient the motility of both populations upward, these simulations show that turbulence actively sorts swimmers by their shape: Elongated organisms move into upwelling regions, while spherical ones move into downwelling regions (Fig. 3, A to C) (*20*, *25*, *26*, *29*). We then carried out simulations for different values of α and computed the average vertical fluid velocity at the location of the cells (Fig. 3D). These simulations predict that a chain growing in length will transition from preferentially sampling flows moving in the opposite direction to their migration to preferentially sampling flows moving in the same direction as their migration. For relatively unstable chains (Ψ = 5 or 10), we find that this transition occurs once chains are two cells in length (α = 0.6), which is in agreement with predictions from an analytical model (*26*), and a recent study used DNS to map out how this process depends on the other nondimensional parameters (*29*). Together, we predict that single cells have to fight against a “headwind” when migrating in turbulence; however, highly elongated chains can experience either a reduced headwind or a “tailwind” that propels them faster in the direction of their migration (Fig. 4B and fig. S3). Similar phenomena (albeit not driven by motility) have been shown to increase the sedimentation of rain droplets and elongated, nonmotile phytoplankton, each of which is preferentially swept into downwelling regions of the flow (*32*–*34*).

### Chain formation often enhances vertical migration through a turbulent water column

The relationship between chain formation and vertical migration is complex: Chain formation reduces stability (Fig. 1) but increases swimming speed (*3*, *6*, *8*, *19*), mean vertical orientation in turbulence (Fig. 2), and preferential motion into flows that favor vertical migration (Fig. 3). While in the preceding sections we have investigated these processes independently, vertical migration ultimately depends on how these mechanisms work together. Here, we use a combination of empirical regressions and theoretical models to estimate how our three nondimensional parameters (Ψ, Φ, and α) vary with both chain length and turbulent intensity, to understand how chain formation affects migration rates across a wide range of conditions.

To estimate how swimming speed, *V*_C_, increases with chain length, we compiled all known experimental measurements of chain swimming speeds from the literature (Fig. 4A). A regression reveals that the swimming speed of a chain $V_{C}^{\left(n\right)}$, normalized by the swimming speed of a single cell $V_{C}^{\left(1\right)}$, follows a power law, $V_{C}^{\left(n\right)}/V_{C}^{\left(1\right)}=n^{\beta }$, where *n* is the number of cells in the chain. All measurements from the literature fall within the envelope formed by β = 0.2 to 0.6, and we thus focused on β = 0.4 as the baseline case, further considering β = 0.2 and β = 0.6 to represent potential variability between species (Fig. 4A). To calculate how the reorientation time scale, *B* = μα_⊥_/(2*h*ρ*g*), increases with chain length, we note that for a sphere, one has α_⊥_ = 6 and, therefore, for a chain, one has *B*^(*n*)^ = *B*^(1)^α_⊥_/6, where α_⊥_ is solely a function of the chain length, *n* (see Supplementary Text) (*35*). We then use typical values from the literature ($V_{C}^{\left(1\right)}=500$ μm/s; *B*^(1)^ = 5 s) to estimate the typical values of $V_{C}^{\left(n\right)}$ and *B*^(*n*)^ of a chain as it grows in length (*2*, *21*–*23*, *28*).

Using these parameterizations, we calculate the nondimensional chain stability number, Ψ, and swimming number, Φ, for different chain lengths, *n*, over the range of turbulent dissipation rates routinely observed in marine systems, ε = 10^−9^ to 10^−6^ W/kg (Fig. 4, B and C) (*36*). This revealed that the mean vertical flow velocity at the centroid of a chain increases as it grows in length, indicating that longer chains have a stronger tendency to move into regions of the flow that transport them along their direction of migration (Fig. 4B). This effect is most pronounced in relatively weak turbulence (ε = 10^−9^ W/kg) where chain formation causes a regime switch, with long chains accumulating in flows moving in the same direction as their migration and spherical cells accumulating in flows moving in the opposite direction (Fig. 4B and fig. S3). We note, however, that elongation can enhance migration even in the absence of a regime switch (i.e., elongation does not necessarily have to make 〈*u_z_*〉 positive; it can also make 〈*u_z_*〉 less negative; Fig. 4B and fig. S3). In relatively strong turbulence (ε = 10^−6^ W/kg), chain swimming speeds are on the same order as the Kolmogorov velocity (Φ ≈ 1), and neither single cells nor chains move appreciably into regions of either downwelling or upwelling. We also observe that the effect of elongation on the mean swimming direction depends on the intensity of turbulence: Chains swimming in relatively weak turbulence (ε = 10^−9^ to 10^−8^ W/kg) can keep more of their motility directed in the direction of migration compared to single cells. In stronger turbulence, the swimming direction of both single cells and chains is more random, although in this case chains have decreased capacity to maintain a vertical orientation (Fig. 4C).

To quantify the combined effect of these three processes—the increased swimming speed, the change in vertical flow velocity sampled by the swimmers, and the change in orientation of chains relative to single cells—we computed the overall vertical migration rate of chains normalized by that of a single spherical cell (Fig. 4D). Using β = 0.4, we find that in relatively weak turbulence (ε = 10^−9^ to 10^−8^ W/kg), chains of two to eight cells can traverse the water column 35 to 130% faster than single cells. Two-cell chains showed enhanced vertical migration rates (10 to 45% faster) across the entire range of turbulent dissipation rates that we tested, indicating that even short chains experience an advantage from chain formation. However, in more intense turbulence, longer chains do not always achieve faster vertical migration: For ε = 10^−7^ to 10^−6^ W/kg, a chain that increases its length beyond two cells reduces its vertical migration rate (Fig. 4D).

While longer chains are well known to swim faster, our results demonstrate that the effect of chain formation on vertical migration depends on the intensity of turbulence. For example, eight-cell chains swim approximately twice as fast as single cells, yet their vertical migration rate is approximately half that of single cells at ε = 10^−6^ W/kg. However, we note that at ε = 10^−6^ W/kg, the vertical migration rate for both single cells and eight-cell chains is already quite weak, being only 14 and 3.6% of their respective swimming speeds (fig. S4). In relatively weak turbulence (ε = 10^−9^ W/kg), both single cells and eight-cell chains migrate through the water column at approximately the same speed at which they swim (fig. S4), giving the faster swimming chains a distinct advantage. These results indicate that chain formation strongly benefits vertical migration in relatively weak turbulence, whereas single cells are potentially more effective migrators when turbulence is relatively strong.

## DISCUSSION

Chain formation markedly reduces the intrinsic stability of cells (Fig. 1E), which would intuitively lead to the expectation of a reduced capacity to migrate through turbulence. In stark contrast to this expectation, we find that this effect can be more than compensated by two phenomena arising from the elongated shape of chains, which, in relatively weak turbulence, drives chains into regions of the flow that aid migration and further allows them to better keep their motility pointed in the direction of their migration. These two processes, along with the greater swimming speed of chains (Fig. 4A; Materials and Methods), allow chains to migrate faster across the water column than single cells whenever turbulence is relatively weak (Fig. 4D). In stronger turbulence, on the other hand, chain formation accelerates the migration of short chains but can retard the migration of longer chains. The turbulent dissipation rate in the ocean varies by several orders of magnitude across time and space (from ε = 10^−10^ to 10^−4^ W/kg). However, the average dissipation rate of the surface ocean is in the order of ε = 10^−7^ W/kg (*36*), which is approximately the value for which we predict that chain formation turns from beneficial to potentially detrimental to vertical migration (Fig. 4D). Thus, we expect that both regimes occur widely in nature.

Because migration is enhanced by chain formation only in some turbulent regimes (Fig. 4D), our simulations suggest that phytoplankton would benefit from the ability to actively measure turbulence levels in their environment and use this information to regulate chain length. Turbulence has been observed to stimulate chain formation in the harmful algal bloom–forming *Alexandrium catenella*: In quiescent conditions, ≈85% of the cells were solitary, whereas in turbulence, the majority of cells were found in eight-cell chains (*17*). These observations suggest that phytoplankton could regulate their length in response to turbulence, in a similar way to how copepod secretions can induce phytoplankton chains to change their length (*3*). While speculative, this behavior would be consistent with previous studies that have shown that phytoplankton can actively sense fluid movement (*10*, *23*, *37*).

The potential migratory benefits of chain formation come with trade-offs: Cells within chains can suffer from increased predation by copepods (*3*) and decreased access to light and nutrients due to interference from clonemates (*4*). It is likely, therefore, that chain formation is used selectively to capitalize on its benefits to migration while minimizing its drawbacks. The rate of downward migration of unicellular phytoplankton species through the water column in the evening is typically 50 to 300% faster than their upward migration in the morning hours, likely owing to gravity, which acts to sediment negatively buoyant phytoplankton cells downward (*2*). Thus, it is interesting to speculate that phytoplankton might preferentially form chains to reduce the longer segment of their commute—the one going upward—rather than their shorter, downward one. This prediction is consistent with field observations of *Cochlodinium polykrikoides*, which indicate that chain formation preferentially occurs during upward migration: While most cells were solitary before their downward migration at dusk, the majority of cells had formed chains by the start of their early morning upward migration (*15*). Elongation-assisted migration might also extend to non–chain-forming species: Cell division in phytoplankton often occurs in the early morning hours so that cells migrating upward are often present in two-cell “chains” (*2*, *38*), which our model suggests would allow them to travel to the surface approximately 45% faster than single cells, provided that ε < 10^−6^ W/kg. Last, we note that single cells of many species of motile phytoplankton have elongated morphologies (e.g., members of the genus *Ceratium* and Fig. 1A) and may benefit from the mechanisms described here.

The marine environment harbors a diverse community of phytoplankton that live under intense predation pressure and competition for finite resources. Our results suggest that chain formation provides cells a simple mechanism to enhance migration through a turbulent water column, which could confer a competitive advantage when light and limiting nutrients do not occur at the same depth. Because of the wide variation in swimming abilities and cell morphologies found within typical phytoplankton communities, this information may prove useful in understanding why one phytoplankton species or genotype is able to dominate over another in the ocean. Because many chain-forming species secrete toxic compounds (*2*, *13*, *14*, *17*), sometimes with toxicity being positively correlated with chain length (*13*), this information may be especially important for understanding the processes that give rise to harmful algal blooms. Beyond pointing to the need to better understand the fundamental interactions between phytoplankton and flow, our results demonstrate that elongated morphologies can confer a distinct advantage to vertically migrating plankton and provide mechanistic insights into how turbulence shapes the composition of phytoplankton communities.

## MATERIALS AND METHODS

### Direct Numerical Simulation (DNS) of turbulent flow

We modeled a homogeneous, isotropic turbulent flow field by direct integration of the Navier-Stokes equations without any closure model. This method, known as DNS, is computationally expensive but yields the most accurate representation of small-scale turbulence possible. The Navier-Stokes equations are given by

$$\rho [\frac{\partial u}{\partial t}+(u\cdot \nabla )u]=-\nabla P+\mu \nabla^{2}u+f$$ (3)

where **u** is the fluid velocity; *P* is the pressure; μ is the dynamic fluid viscosity; **f** is a zero-mean, temporally uncorrelated Gaussian forcing to sustain statistically steady turbulence; and flow is incompressible such that **∇** · **u** = 0. The computational domain is a cubic box with triply periodic boundary conditions. The solution of Eq. 3 is time-advanced using a second-order Adams-Bashforth scheme, while the Poisson equation for the pressure is solved with a spectral method. The unsteady forcing is spread over a narrow shell of small wave numbers to generate large-scale flows. To eliminate aliasing errors, we used the 2/3 dealiasing technique, which sets the largest 1/3 of all wave numbers to zero after each computation of the nonlinear terms in the Navier-Stokes equations such that the largest resolved wave number is $k_{\text{max}}=\frac{1}{3}M^{1/3}$. We used a Fourier pseudospectral code to simulate the flow using *M* = 128^3^ mesh points, which ensures that the smallest eddies in the flow are adequately resolved (*k*_max_η_K_ ≤ 1).

At steady state, the average rate of kinetic energy dissipation per unit volume, ε, is equal to the average rate of energy injected into the system through the large-scale forcing. The total dissipation rate can be directly calculated by integrating the dissipation spectrum *D*(*k*) over the entire range of wave numbers

$$\varepsilon =\int_{0}^{k_{\text{max}}}D(k)\;\mathrm{dk}=2\nu \int_{0}^{k_{\text{max}}}k^{2}E(k)\mathrm{dk}$$ (4)

where *E*(*k*) is the energy spectrum.

A measure of the diversity of length scales within the turbulent flow is given by the Taylor Reynolds number, *Re*_λ_ = *u*_rms_λ/ν, where *u*_rms_ is the root mean square flow velocity, ν is the kinematic viscosity, and $\lambda =\sqrt{15\nu u_{\text{rms}}^{2}/\varepsilon }$ is the Taylor length scale. Our simulations used *Re*_λ_ = 62, which previous work has shown to be sufficient to resolve the interactions of gyrotactic swimmers with turbulent flow (*20*).

### Individual-based model of gyrotaxis

To resolve how phytoplankton chains migrate through the DNS of turbulence, we initialized our computational domain with 100,000 Lagrangian swimmers after the turbulent flow had reached a statistical steady state. The initial positions of swimmers were randomly (Poisson) distributed in space, and their initial swimming directions, **p**, were randomly distributed on a unit sphere. At each time step of the simulation, the fluid flow properties (**u**, **ω**, and **E**) at the location of each swimmer were calculated using trilinear interpolation, and the swimmer orientation and position were integrated using a second-order Adams-Bashforth scheme. All of our analyses were performed after swimmers had reached a statistically steady distribution, which, in our simulations, corresponds to one to two integral time scales, which is the turnover time of the largest eddies in the flow (equivalent to approximately 40 Kolmogorov time scales). We verified the convergence of our analyses using separate tests that integrated the trajectories of 300,000 swimmers until they reached a statistical steady-state distribution.

Our simulations modeled cells as point-like particles, which previous studies have found to be a good approximation provided that the particles are smaller than approximately 2η_K_ or 3η_K_ (*39*, *40*). The Kolmogorov scale for the largest turbulent dissipation rate considered here (ε = 10^−6^ W kg^−1^) is η_K_ = 1 mm, suggesting that the point approximation is valid because the longest chains are typically less than few hundred micrometers in length (*3*, *6*, *8*, *14*, *16*, *17*, *19*). We also note that Eqs. 1 and 2 do not include the effect of phytoplankton inertia, owing to their small size and density close to that of the water in which they live (*33*). Furthermore, our model also assumes that phytoplankton chains are rigid and do not bend in flow. While previous studies have directly measured the stiffness of nonmotile diatom chains (*41*), we are not aware of any studies that directly measure the stiffness of motile phytoplankton chains or how bending might affect their motility.

### Estimating how swimming speed changes as a function of chain length

Phytoplankton chains have been widely reported to increase their swimming speed as the number of cells increases (*3*, *6*, *8*, *19*). This finding has been attributed to the fact that adding more cells to a chain increases its propulsive force more than it increases its hydrodynamic drag (*8*). While a regression of experimental data from the literature captures this process, we also considered two theoretical models (green lines in Fig. 4A).

Similar to a previous study (*8*), both of our models assume that the propulsive force of a chain, $F_{P}^{\left(n\right)}$, scales linearly with the number of cells, *n*, such that $F_{P}^{\left(n\right)}=nF_{P}^{\left(1\right)}$, where $F_{P}^{\left(1\right)}$ is the propulsive force generated by a single cell. This assumes that the cells within a chain do not interfere with one another’s propulsion within a chain; thus, this formulation may represent an upper bound (*8*). The drag force on a chain, *F*_D_, can be modeled using Stokes law as $F_{D}^{\left(n\right)}=3\pi \mu V_{C}^{\left(n\right)}d_{e}K$, where μ is the dynamic fluid viscosity, $V_{C}^{\left(n\right)}$ is the swimming speed, *K* is a shape correction factor, and *d_e_* is the equivalent diameter of the chain.

We considered two different models for *F*_D_, which differ in their formulation of *K* and *d_e_*.The first model approximates a chain as a row of *n* rigidly attached spheres of diameter *d*, where *d_e_* = *n*^1/3^*d* is the diameter of a single sphere whose volume is equal to the sum of the volume of all of the spheres within a chain and *K* is a correction factor that has been quantified via numerical simulations and with experiments (*42*, *43*). Equating the drag force on the chain of spheres with the propulsive force yields $V_{C}^{\left(n\right)}=F_{P}^{\left(1\right)}n/3\pi \mu d_{e}K$. This expression can be simplified by considering the swimming speed of a single cell of diameter *d*, which exerts a propulsive force $F_{P}^{\left(1\right)}$ that is balanced by a drag force of $F_{D}=3\pi \mu V_{C}^{\left(1\right)}d$, which yields $F_{P}^{\left(1\right)}=3\pi \mu V_{C}^{\left(1\right)}d$. Substitution thus yields a chain swimming speed of $V_{C}^{\left(n\right)}=n^{2/3}V_{C}^{\left(1\right)}/K$, and dividing both sides of this expression by the Kolmogorov velocity allows us to nondimensionalize, yielding Φ^(*n*)^ = *n*^2/3^Φ^(1)^/*K*.

The second model approximates a chain as a prolate spheroid whose aspect ratio is equal to the number of cells within the chain, *n*, and *d_e_* is the diameter of the spheroid’s minor axis. Using the same process as above yields Φ^(*n*)^ = *n*Φ^(1)^/*K*, where the correction factor *K* is given by (*44*)

$$K=\frac{\frac{4}{3}(n^{2}-1)}{\frac{2n^{2}-1}{{\left(n^{2}-1\right)}^{0.5}}\text{log}\;[n+{\left(n^{2}-1\right)}^{0.5}]-n}$$

For convenience, we provide the numerical values of *K* for both models in table S1.

### Resolving how the distance between a chain’s center of mass and center of buoyancy varies with chain length

In this section, we used a simple model to show that *h*, the distance between a chain’s center of buoyancy, $z_{\text{cb}}^{\left(n\right)}$, and center of mass, $z_{\text{cm}}^{\left(n\right)}$, is the same as that of a solitary phytoplankton cell and does not vary with chain length, *n*. The center of buoyancy of a spherical individual cell (*n* = 1) occurs at its geometrical center such that $z_{\text{cb}}^{\left(1\right)}=d/2$, while *h* scales with cell diameter *d* (*21*) such that $h^{\left(1\right)}=z_{\text{cm}}^{\left(1\right)}-z_{\text{cb}}^{\left(1\right)}=\alpha d$. Therefore, via substitution, we obtained for a single cell that

$$z_{\text{cm}}^{\left(1\right)}=\alpha d+d/2$$

To find the center of mass of a chain, we summed the product of the mass *m* and *z*_cm_ for each of its constituent cells and then divided by the total mass of the chain. For a chain of two cells (fig. S5), we therefore have

$$z_{\text{cm}}^{\left(2\right)}=\frac{z_{\text{cm}}^{\left(1\right)}m+(d+z_{\text{cm}}^{\left(1\right)})m}{2m}=z_{\text{cm}}^{\left(1\right)}+d/2$$

By definition, the separation between the center of mass and center of buoyancy for a two-cell chain is given by

$$h^{\left(2\right)}=z_{\text{cm}}^{\left(2\right)}-z_{\text{cb}}^{\left(2\right)}$$

Noting that $z_{\text{cb}}^{\left(2\right)}=d$ by symmetry and substituting the previous result for $z_{\mathrm{cm}}^{\left(1\right)}$, we obtained

$$h^{\left(2\right)}=\alpha d$$

which shows that a single cell and a two-cell chain have the same *h*.

Using the same logic, we then expanded this analysis for a chain of generic length *n*, which yields

$$z_{\text{cm}}^{\left(n\right)}=\frac{{\mathrm{nz}}_{\text{cm}}^{\left(1\right)}+\sum_{i=1}^{n-1}\mathrm{id}}{n}=z_{\text{cm}}^{\left(1\right)}+d(n-1)/2$$

and consequently

$$h^{\left(n\right)}=z_{\text{cm}}^{\left(n\right)}-z_{\text{cb}}^{\left(n\right)}=z_{\text{cm}}^{\left(1\right)}+d(n-1)/2-\mathrm{nd}/2=\alpha d$$

Thus, a chain has the same *h* as each of its constituent cells.

## Supplementary Material

http://advances.sciencemag.org/cgi/content/full/5/10/eaaw7879/DC1

Download PDF

Chain formation can enhance the vertical migration of phytoplankton through turbulence

## SUPPLEMENTARY MATERIALS

Supplementary material for this article is available at http://advances.sciencemag.org/cgi/content/full/5/10/eaaw7879/DC1 Fig. S1. Elongation drives patchiness in the distribution of motile cells swimming in turbulence, and this peaks at intermediate swimming speeds. Fig. S2. Elongation causes the distribution of gyrotactic swimmers to become less patchy at small values of ψ and more patchy at large values of ψ. Fig. S3. Spherical gyrotactic swimmers (α = 0) preferentially sample flows that move in the direction opposite to that of their motility. Fig. S4. Stronger turbulence impedes vertical migration, increasing the amount of time that chains require to traverse a water column. Fig. S5. A simple model of bottom heaviness reveals that the distance between the center of mass and center of buoyancy of a chain is independent of chain length. Table S1. The drag force shape correction factors, *K*, for the two models used to estimate chain swimming speed.

## Appendix

View/request a protocol for this paper from *Bio-protocol*.
