# Supplementary material for: Chain formation can enhance the vertical migration of phytoplankton through turbulence
Source: Sci Adv. 2019 Oct 16;5(10):eaaw7879. doi: 10.1126/sciadv.aaw7879 (PMC6795514; doi:10.1126/sciadv.aaw7879)
Supplement: http://advances.sciencemag.org/cgi/content/full/5/10/eaaw7879/DC1 [file supp_5_10_eaaw7879__index.html]

Science Advances | Science AdvancesAAASSearchScience AdvancesMenu

## Supplementary Materials

**This PDF file includes:**

- Fig. S1. Elongation drives patchiness in the distribution of motile cells swimming in turbulence, and this peaks at intermediate swimming speeds.
- Fig. S2. Elongation causes the distribution of gyrotactic swimmers to become less patchy at small values of ψ and more patchy at large values of ψ.
- Fig. S3. Spherical gyrotactic swimmers (α = 0) preferentially sample flows that move in the direction opposite to that of their motility.
- Fig. S4. Stronger turbulence impedes vertical migration, increasing the amount of time that chains require to traverse a water column.
- Fig. S5. A simple model of bottom heaviness reveals that the distance between the center of mass and center of buoyancy of a chain is independent of chain length.
- Table S1. The drag force shape correction factors, *K*, for the two models used to estimate chain swimming speed.

Download PDF

**Files in this Data Supplement:**

- Adobe PDF - aaw7879\_SM.pdf
