# Supplementary material for: Chain formation can enhance the vertical migration of phytoplankton through turbulence
Source: Sci Adv. 2019 Oct 16;5(10):eaaw7879. doi: 10.1126/sciadv.aaw7879 (PMC6795514; doi:10.1126/sciadv.aaw7879)
Supplement: Download PDF [file aaw7879_SM.pdf]

Science Advances
AAAS

advances.sciencemag.org/cgi/content/full/5/10/eaaw7879/DC1

Supplementary Materials for

Chain formation can enhance the vertical migration of phytoplankton through turbulence

Salvatore Lovecchio, Eric Climent, Roman Stocker, William M. Durham*

*Corresponding author. Email: w.m.durham@sheffield.ac.uk

Published 16 October 2019, *Sci. Adv.* **5**, eaaw7879 (2019)
DOI: 10.1126/sciadv.aaw7879

This PDF file includes:

Fig. S1. Elongation drives patchiness in the distribution of motile cells swimming in turbulence, and this peaks at intermediate swimming speeds.
Fig. S2. Elongation causes the distribution of gyrotactic swimmers to become less patchy at small values of ψ and more patchy at large values of ψ.
Fig. S3. Spherical gyrotactic swimmers ($\alpha = 0$) preferentially sample flows that move in the direction opposite to that of their motility.
Fig. S4. Stronger turbulence impedes vertical migration, increasing the amount of time that chains require to traverse a water column.
Fig. S5. A simple model of bottom heaviness reveals that the distance between the center of mass and center of buoyancy of a chain is independent of chain length.
Table S1. The drag force shape correction factors, *K*, for the two models used to estimate chain swimming speed.

SUPPLEMENTARY FIGURES

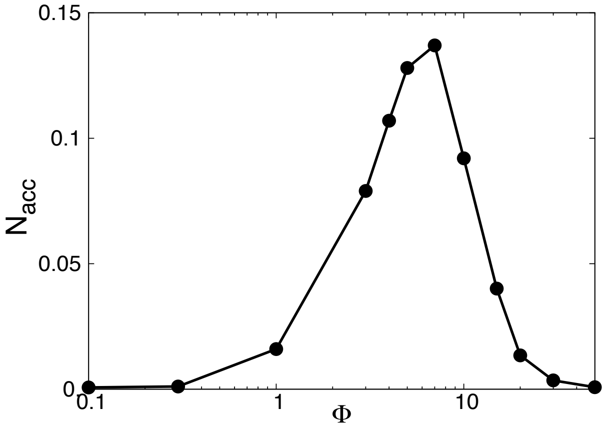

Fig. S1. Elongation drives patchiness in the distribution of motile cells swimming in turbulence, and this peaks at intermediate swimming speeds. While gyrotaxis can drive strong patchiness in the distribution of phytoplankton (20), it has been recently demonstrated that elongation can also drive patchiness in the distribution of motile cells (25, 26, 29, 31). Here we performed simulations of highly elongated, non-gyrotactic phytoplankton cells ($\alpha = 1$ and $\Psi = \infty$) in homogeneous isotropic turbulence, and quantified the level of patchiness using the normalized box probability index, N_{acc} (ref. (20)). This analysis unexpectedly reveals that the patchiness driven by elongation peaks at intermediate swimming speeds ($\Phi \approx 7$). This finding stands in stark contrast with the patchiness generated by spherical gyrotactic cells, which increases monotonically over the same range of Φ (ref. (20)). Briefly, N_{acc} is calculated by subdividing our computational domain into equally spaced boxes and counting the number of cells within each box. When the distribution of cells is patchy, some boxes have a large number of cells whilst others have relatively few. Thus the standard deviation of the number of cells in each box, σ, increases as the degree of patchiness increases. The measured standard deviation is normalized by the standard deviation expected for a random (Poisson) distribution of cells, $\sigma_P = \sqrt{\mu_{cells}}$, where μ_{cells} is the mean number of cells within the boxes. This normalization yields $N_{acc} = \frac{\sigma - \sigma_P}{\mu_{cells}}$. Thus, $N_{acc} = 0$ corresponds to a random (Poisson) distribution of cells, whilst larger N_{acc} indicates a more patchy distribution. Since the value of N_{acc} depends on the size of the boxes used to subdivide the computational domain, we computed the particle number density distribution for many different sizes of boxes and used the largest value of N_{acc} as a measure of accumulation. The box size that yields the largest N_{acc} gives a rough estimate of the length scale of the clusters, which in our simulations was $\approx 18\eta_K$.

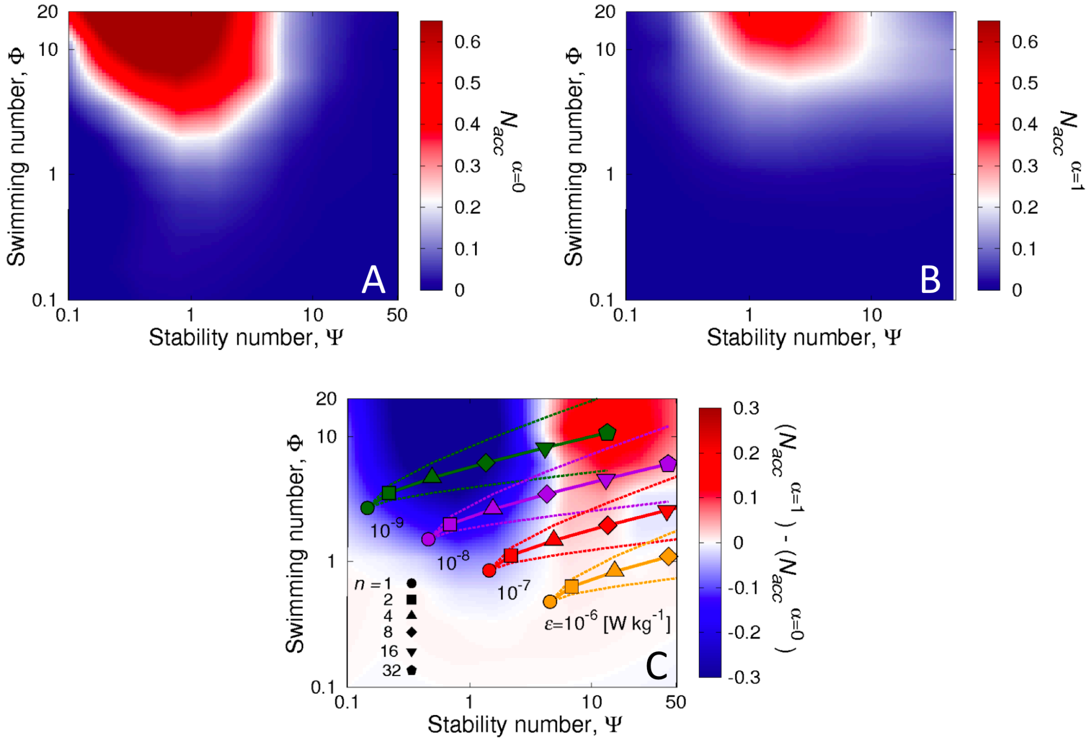

Fig. S2. **Elongation causes the distribution of gyrotactic swimmers to become less patchy at small values of Ψ and more patchy at large values of Ψ.** We used the normalized box probability index, N (see fig. S1), to quantify how elongation effects the clustering of cells as Ψ and Φ changes. In panels **(A)** and **(B)**, we show N_{acc} for spherical ($\alpha = 0$) and highly elongated cells ($\alpha = 1$), respectively, over a range of different Ψ and Φ values. Then, in panel **(C)** we show the change in N_{acc} induced by elongation, where negative (positive) values indicate that elongation reduces (increases) the amount of patchiness. This analysis reveals that elongation can either reduce or increase the amount of patchiness depending on the position in [Ψ, Φ] space. While patchiness reaches a maximum at intermediate values of Ψ for both spherical and elongated gyrotactic swimmers, the position of this maximum occurs at larger values of Ψ for elongated swimmers.

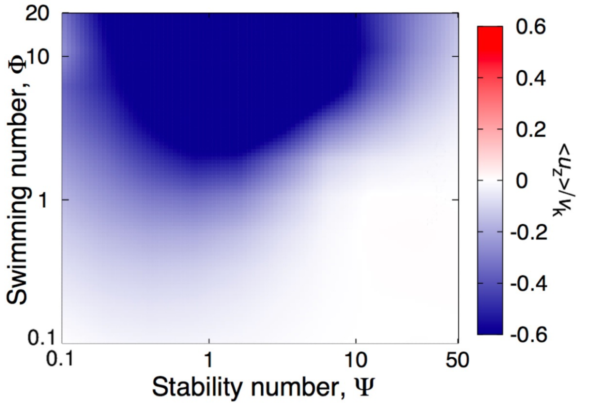

Fig. S3. **Spherical gyrotactic swimmers ($\alpha = 0$) preferentially sample flows that move in the direction opposite to that of their motility.** Here we show the mean vertical velocity sampled by a population of upward swimming spherical cells normalized by Kolmogorov velocity, $\langle u_z \rangle / V_{\mathrm{K}}$. In contrast with highly elongated gyrotactic swimmers ($\alpha = 1$), which can preferentially sample vertical flows either in the upwards or downwards direction depending on their position in $[\Psi, \Phi]$ parameter space (Fig. 4B), spherical gyrotactic cells preferentially sample only the downwelling regions of flow, such that $\langle u_z \rangle / V_{\mathrm{K}} < 0$. The scale of the colormap is the same as that in Fig. 4B.

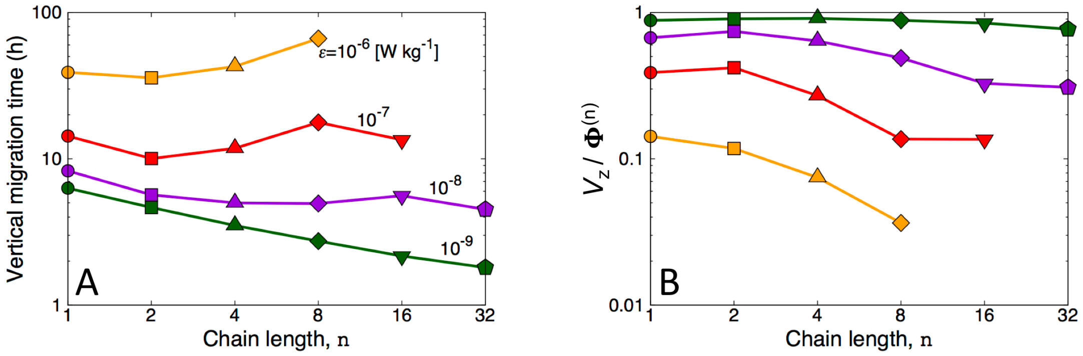

Fig. S4. Stronger turbulence impedes vertical migration, increasing the amount of time that chains require to traverse a water column. In panel **(A)**, we show the amount of time (in hours) required for chains to migrate through 10 meters of water column. This reveals that in relatively strong turbulence ($\varepsilon = 10^{-6}$ W/kg) chains require nearly ten times longer to migrate over a fixed distance compared to that in weaker turbulence ($\varepsilon = 10^{-9}$ W/kg). In Panel **(B)**, we show the overall vertical migration rate of chains scaled by their swimming speed, $V_z/\Phi^{(n)}$. This shows that in relatively weak turbulence ($\varepsilon = 10^{-9}$ W/kg), cells can vertically migrate at nearly the same speed at which they swim $V_z/\Phi^{(n)} \approx 1$, whereas in stronger turbulence cells are only capable of vertically migrating at a small fraction of their swimming speeds $V_z/\Phi^{(n)} \ll 1$. We note that these analyses measure the total vertical migration rate, which is a function of both the mean orientation of cells ($\langle p_z \rangle$, Fig. 4C) and the mean vertical flow sampled by the population ($\langle u_z \rangle$, Fig. 4B).

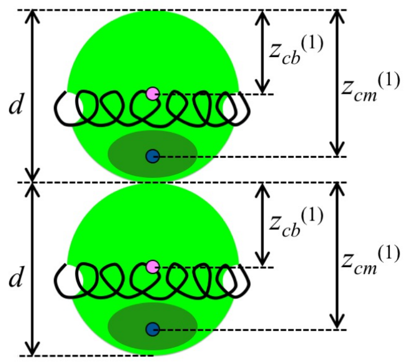

Fig. S5. A simple model of bottom heaviness reveals that the distance between the center of mass and center of buoyancy of a chain is independent of chain length. Here we present a sketch of a two-cell chain to support the calculations shown in the *Materials and Methods* section titled "Resolving how the distance between a chain's center of mass and center of buoyancy varies with chain length".

SUPPLEMENTARY TEXT

Computation of B for phytoplankton chains. To understand how the gyrotactic reorientation timescale varies as chains grow longer, we used a model that assumes phytoplankton chains behave as prolate spheroids with an aspect ratio equal to n, the number of cells within the chain. The gyrotactic reorientation timescale for a prolate spheroid is given by $B = \mu\alpha_\perp/(2h\rho g)$ where $\alpha_\perp$ is the dimensionless resistance coefficient for rotation about an axis perpendicular to $\mathbf{p}$ (the direction of swimming), which is defined as (35):

$$\alpha_\perp = \frac{2(n^2+1)}{3(n^2-1)}\alpha_5,$$

where,

$$\alpha_1 = \frac{1}{I_1}\left(1 + \frac{L_1}{L_2} - \frac{2I_1}{I_2}\right),$$

$$\alpha_2 = -\frac{1}{I_1}\left(1 - \frac{I_1}{I_2}\right),$$

$$\alpha_3 = \frac{1}{I_1},$$

$$\alpha_4 = \frac{1}{3I_1}\left(1 - \frac{L_1}{L_2}\right),$$

$$\alpha_5 = \frac{6(n^4-1)}{2n^2I_2 + (n^2+1)^2L_2},$$

$$I_1 = \frac{n^2(2n^2-5+3\gamma)}{2(n^2-1)^2},$$

$$I_2 = \frac{(n^2+1)(n^2+2-3n^2\gamma)}{(n^2-1)^2},$$

$$L_1 = \frac{n^2[2n^2+1-\gamma(4n^2-1)]}{4(n^2-1)^2},$$

$$L_2 = I_1 - 2L_1,$$

$$\gamma = \frac{\cosh^{-1} n}{n\sqrt{(n^2-1)}}.$$

SUPPLEMENTARY TABLE

Table S1. The drag force shape correction factors, K, for the two models used to estimate chain swimming speed.

number of cells	K (chain of spheres)	K (prolate spheroid)
1	1.00	1.00
2	1.02	1.20
3	1.07	1.40
4	1.11	1.60
5	1.16	1.78
6	1.20	1.97
7	1.23	2.14
8	1.27	2.31

Table I: Correction coefficient, K, for two different models of the drag on a chain
